# Lipopolysaccharide shock reveals the immune function of indoleamine 2,3-dioxygenase 2 through the regulation of IL-6/stat3 signalling

**DOI:** 10.1038/s41598-018-34166-4

**Published:** 2018-10-29

**Authors:** Yasuko Yamamoto, Wakana Yamasuge, Shinjiro Imai, Kazuo Kunisawa, Masato Hoshi, Hidetsugu Fujigaki, Akihiro Mouri, Toshitaka Nabeshima, Kuniaki Saito

**Affiliations:** 1 Department of Disease Control and Prevention, Fujita Health University Graduate School of Health Sciences, Toyoake, 470–1192 Japan; 2 School of Bioscience and Biotechnology, Tokyo University of Technology, Hachioji, 192–0982 Japan; 3 Advanced Diagnostic System Research Laboratory, Fujita Health University Graduate School of Health Sciences, Toyoake, 470–1192 Japan; 4 Department of Regulatory Science, Fujita Health University Graduate School of Health Sciences, Toyoake, 470–1192 Japan; 5 Japanese Drug Organization of Appropriate Use and Research, Nagoya, 468-0069 Japan; 6 Aino University, Ibaraki, 567-0012 Japan; 7 Human Health Sciences, Graduate School of Medicine and Faculty of Medicine, Kyoto University, Kyoto, 606–8507 Japan

## Abstract

Indoleamine 2,3-dioxygenase 2 (Ido2) is a recently identified catalytic enzyme in the tryptophan-kynurenine pathway that is expressed primarily in monocytes and dendritic cells. To elucidate the biological role of Ido2 in immune function, we introduced lipopolysaccharide (LPS) endotoxin shock to Ido2 knockout (Ido2 KO) mice, which led to higher mortality than that in the wild type (WT) mice. LPS-treated Ido2 KO mice had increased production of inflammatory cytokines (including interleukin-6; IL-6) in serum and signal transducer and activator of transcription 3 (stat3) phosphorylation in the spleen. Moreover, the peritoneal macrophages of LPS-treated Ido2 KO mice produced more cytokines than did the WT mice. By contrast, the overexpression of Ido2 in the murine macrophage cell line (RAW) suppressed cytokine production and decreased stat3 expression. Finally, RAW cells overexpressing Ido2 did not alter nuclear factor κB (NF-κB) or stat1 expression, but IL-6 and stat3 expression decreased relative to the control cell line. These results reveal that Ido2 modulates IL-6/stat3 signalling and is induced by LPS, providing novel options for the treatment of immune disorders.

## Introduction

Indoleamine 2,3-dioxygenase 1 (Ido1) is an enzyme that catalyses the oxidation of tryptophan (Trp) to kynurenine (KYN) in the first step of the kynurenine pathway^1^. Ido1 is also an immune modulator in several types of immune cells, such as dendritic cells (DCs) and macrophages^2^. Metabolic changes in the kynurenine pathway due to Ido1 induce T cell apoptosis and T-reg proliferation^3–5^. Because of these immune modulating functions, Ido1 plays important roles in various pathophysiological processes, such as antimicrobial and antitumour defence, neuropathology and immune regulation^3,4,6–11^.

Indoleamine 2,3-dioxygenase 2 (Ido2) is an isoform of Ido1 that was discovered recently^12–14^. The genes encoding these two isoforms are arranged in tandem on the same chromosome across mammals, suggesting that these genes arose via gene duplication^12,14–16^. In addition, their expression patterns differ because of various homeostatic and inflammatory conditions. For example, Ido1 expression is detected in the colon and epididymis, but Ido2 is expressed predominantly in the liver, kidney and epididymis in mice^12,17,18^. Moreover, proinflammatory cytokines in epithelial cells, macrophages and DCs induce Ido1 but not Ido2^19^. Although the induction of Ido2 by proinflammatory cytokines is controversial, a recent study reported the expression of Ido2 in DCs and monocytes. The expression of Ido2 in immune cells suggests that Ido2 contributes to immune function. Recent studies also have revealed that Ido2 modulates immune function in autoantibody^20,21^ and T-reg production^22,23^. However, the biological role of Ido2 in immune function is unclear.

In this study, we investigated the role of Ido2 in the immune response using a lipopolysaccharide (LPS)-induced endotoxin shock model. We demonstrated that an Ido2 knockout (KO) exacerbates the effects of LPS in mice. Indeed, we found strong upregulation of inflammatory cytokine production in the macrophages of Ido2 KO mice following LPS stimulation via the Toll-like receptor 4 (TLR4) and nuclear factor κB (NF-κB) signalling pathways. Furthermore, gene array analysis in the RAW murine macrophage cell line expressing Ido2 (RAW-Ido2) showed that Ido2 overexpression altered cytokine signalling. There was no effect on the expression of NF-κB or signal transducer and activator of transcription 1 (stat1) in RAW cells overexpressing Ido2, but the expression of interleukin (IL)−6, signal transducer and activator of transcription 3 (stat3), suppressor of cytokine signalling 1 (SOCS1) and suppressor of cytokine signalling 3 (SOCS3) decreased. Thus, we show that Ido2 directly contributes to cytokine production by modulating IL-6/stat3 cytokine signalling.

## Results

### Ido2 KO mice are highly sensitive to LPS-induced endotoxin shock

**Figure 1:**
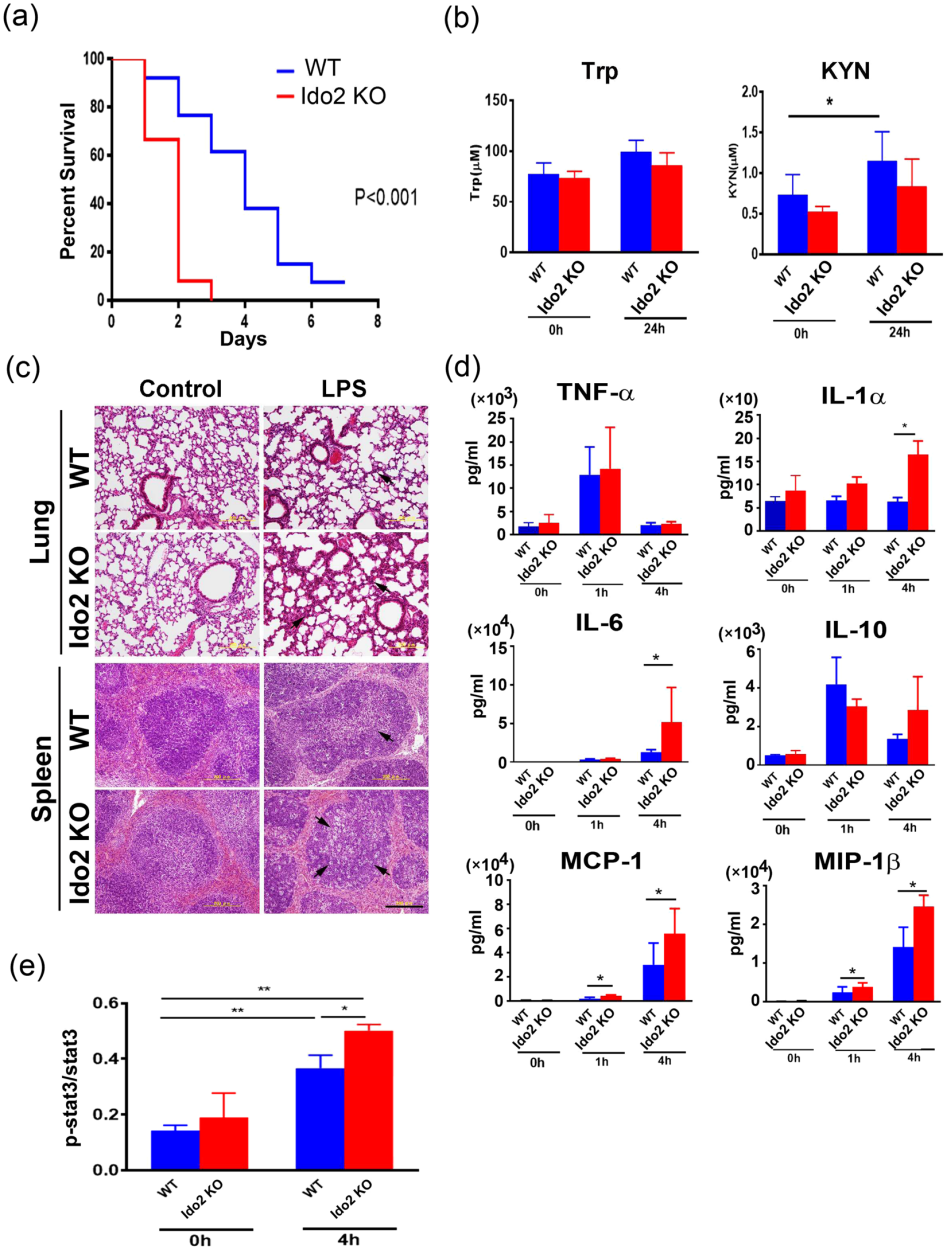
Ido2 KO mice are highly sensitive to LPS-induced endotoxin shock. (**a**) Kaplan–Meier plot of age-matched WT (*n* = 13) and Ido2 KO mice (*n* = 12) treated with LPS (15 mg/kg). Mortality was assessed for 7 days. Statistical analyses were performed using the log-rank test: **p < 0.001 between WT and Ido2 KO mice. (**b**) Serum concentration of Trp and KYN in WT and Ido2 KO mice 24 h after LPS administration (*n* = 5 each). (**c**) Representative images from H&E staining of lung and spleen sections from WT and Ido2 KO mice 24 h after LPS administration. Scale bar, 200 μm. (**d**) Cytokine concentrations in serum were determined with Bioplex. Samples were taken at 1 and 4 h after LPS administration in WT and Ido2 KO mice (*n* = 5 each). (**e**) Protein expression of phospho-stat3 was examined in the spleen of WT and Ido2 KO mice 4 h after LPS administration (*n* = 5 each). Data are presented as the mean ± SD. *p < 0.05.

To elucidate the biological function of Ido2 in the immune response, we used an LPS-induced endotoxin shock model. After injection of the lethal dose of LPS (15 mg/kg), mortality was examined in Ido2 KO mice. Ido2 KO mice had higher mortality than did the WT mice (*p* < 0.001) (Fig. 1a). Twenty-four hours after LPS stimulation, the concentration of serum Trp and KYN was examined, and histological analysis of the lung and spleen was performed. There was no difference in serum Trp concentration between WT mice and Ido2 KO mice, but the increase in KYN concentration found in WT mice was not observed in Ido2 KO mice (Fig. 1b). In Ido2 KO mice, histological analyses of the lung and spleen revealed that LPS-induced endotoxin shock increased inflammatory cell invasion and tissue injury severity compared with WT mice (Fig. 1c). Immunohistochemical analysis with anti- Ido2 antibody revealed an increase in Ido2-positive immune cells in the lung and spleen when treated with LPS (Supplemental Fig. 1). After LPS stimulation (1 and 4 h), both WT and Ido2 KO mice showed an increases in serum levels of inflammatory cytokines, including tumour necrosis factor-α (TNF-α), IL-1α, IL-6, IL-10, monocyte chemoattractant protein-1 (MCP-1) and macrophage inflammatory protein (MIP)−1β (Fig. 1d). TNF-α induction was nearly the same in both WT and Ido2 KO mice at 1 and 4 h, but the production of IL-1α, IL-6, MCP-1 and MIP-1β in Ido2 KO mice was significantly higher than that in WT mice at 4 h. Ido2 KO mice produced more IL-6 than did the WT mice at 4 h (Fig. 1d). These results suggest that the induction of inflammatory cytokines may be the cause of the higher mortality in Ido2 KO mice after LPS stimulation. Furthermore, the rate of LPS-induced stat3 phosphorylation increased in the spleen of Ido2 KO mice compared with WT mice at 4 h (Fig. 1e). Ido2 KO mice showed an accelerated rate of inflammatory cytokine production that worsened the pathophysiology of the LPS stimulus.

### The peritoneal macrophages of Ido2 KO mice upregulate cytokine production following LPS stimulus

Cytokine production from immune cells is an important process in the inflammatory response. Given that Ido2 KO mice showed uncontrolled cytokine production following LPS-induced endotoxin shock, Ido2 may affect cytokine production in immune cells. Among the immune cells, macrophages are key regulators of the immune response. Therefore, we examined the mRNA expression levels of Ido1 and Ido2 in the peritoneal macrophages of WT mice following LPS stimulation, and indeed, the peritoneal macrophages from WT mice had induced the mRNA expression of Ido1 and Ido2 (Supplemental Fig. 2).

**Figure 2:**
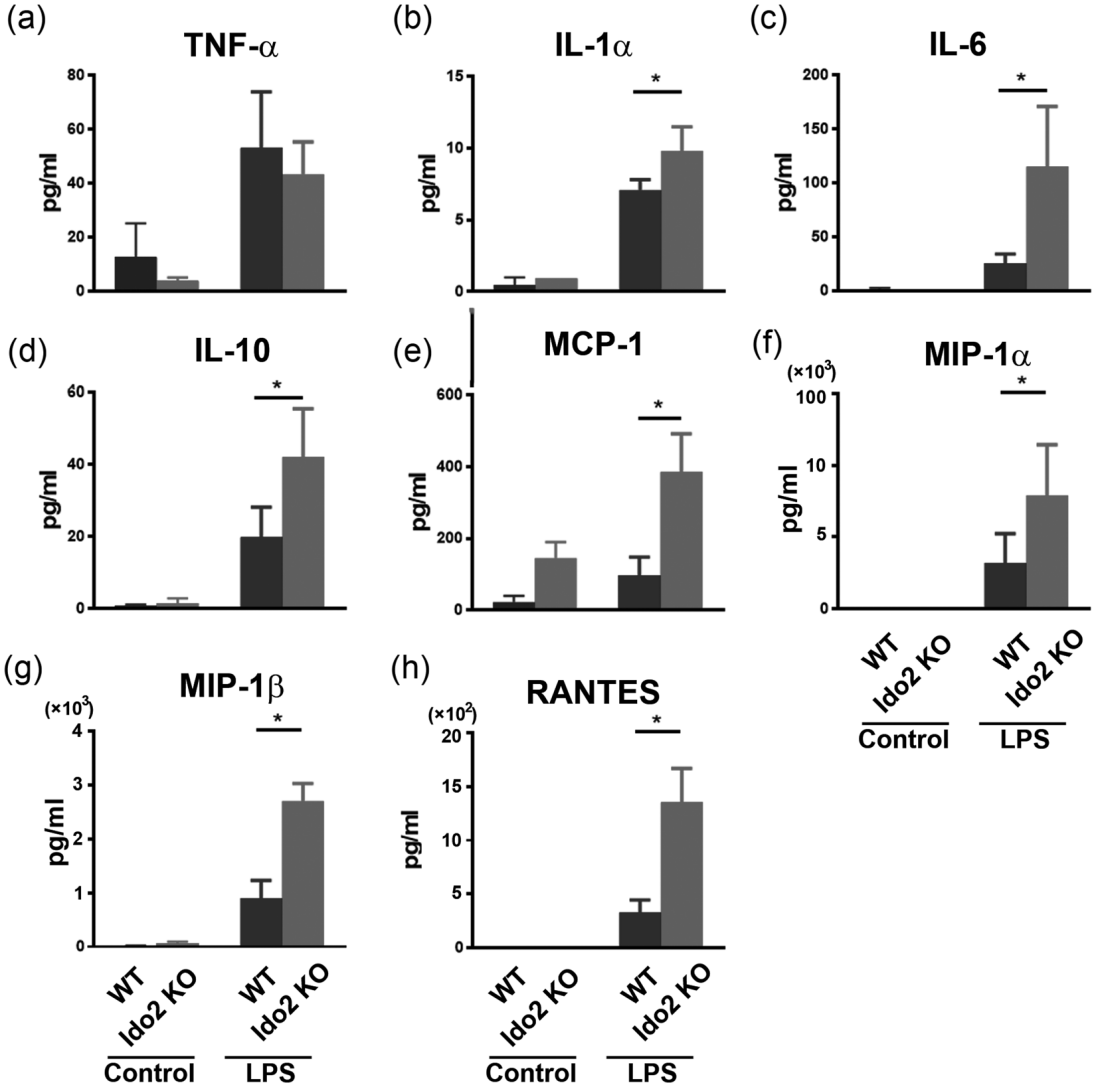
The peritoneal macrophages of Ido2 KO mice upregulate cytokine production following LPS stimulus. The inflammatory cytokine and chemokine levels of peritoneal macrophages in WT and Ido2 KO mice 24 h after LPS treatment (100 ng/mL) were examined with Bioplex. The Ido2 KO accelerated the production of various cytokines and chemokines. Data are presented as the mean ± SD (*n* = 5–7). *p < 0.05.

To examine Ido2 function in macrophages induced by LPS, peritoneal macrophages from WT and Ido2 KO mice were cultured with LPS, and the concentration of cytokines in the conditioned media was measured. Peritoneal macrophages from Ido2 KO mice significantly upregulated the production of cytokines, such as IL-1α, IL-6, IL-10, MCP-1, MIP-1a, MIP-1b and regulated on activation normal T cell expressed and secreted (RANTES) compared with WT mice (Fig. 2).

To clarify the effective immune cell type, we tested whether T cells played a role in cytokine production in WT and Ido2 KO mice. Because IL-2 and IL-4 are the most secreted cytokines from T cells following stimulus, we measured the levels of IL-2 and IL-4 in conditioned medium from the T cells of WT and Ido2 KO mice. The Ido2 KO T cells did not show any difference in cytokine production compared with those of WT mice (Supplemental Fig. 3). These results reveal that the macrophages are the effective immune cells in Ido2 KO mice that upregulate cytokine production. On the other hand, the macrophages of Ido1 KO mice did not show a significant difference in cytokine production (IL-6, MCP-1, MIP-1a and MIP-1b) compared with those of WT mice (Supplemental Fig. 4), but the expression of Ido1 mRNA was induced by LPS (Supplemental Fig. 2).

### Cytokine signalling is blocked due to overexpression of Ido2 in RAW cells

**Figure 3:**
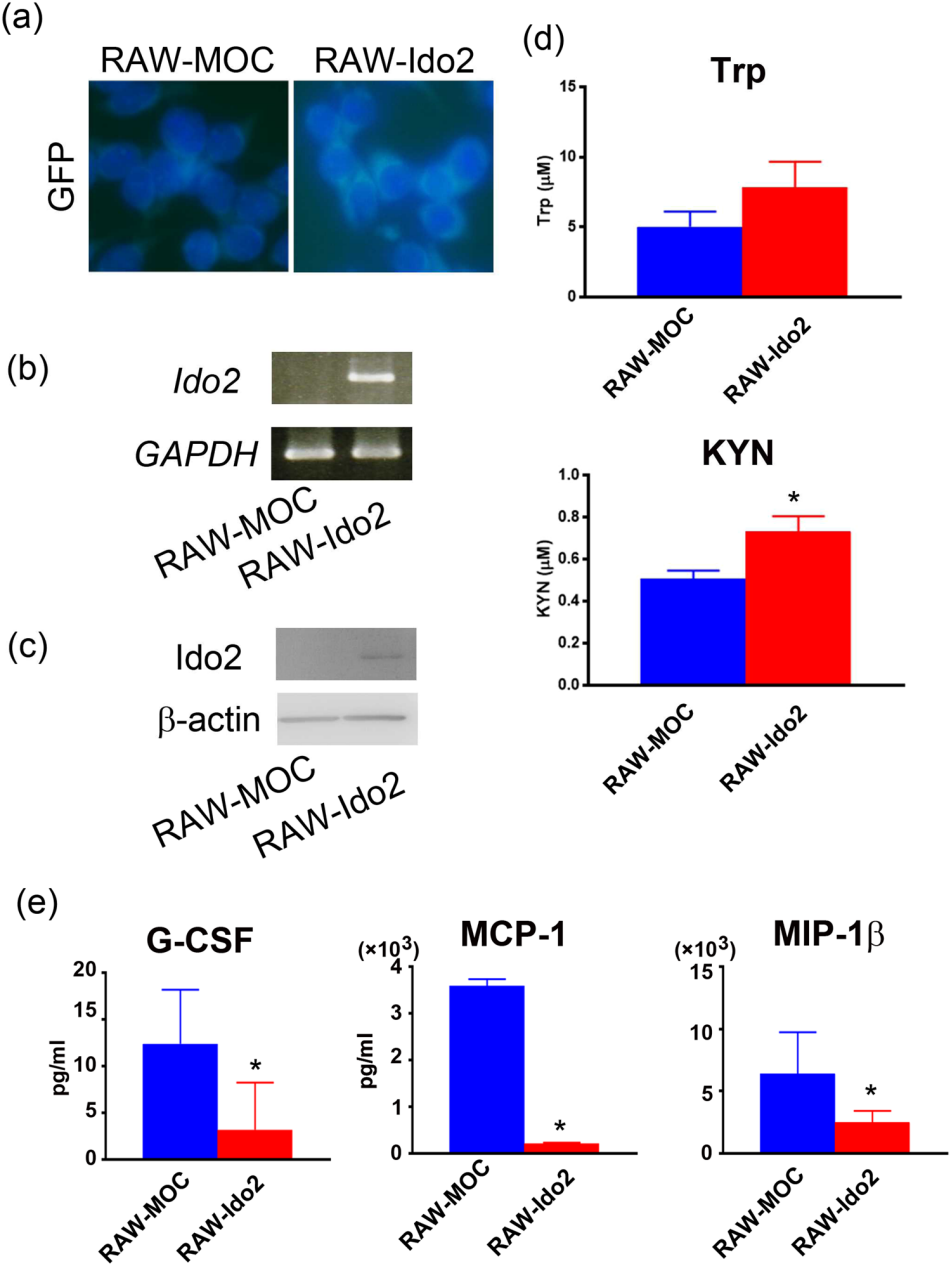
The overexpression of Ido2 in RAW cells blocks cytokine signalling. (**a**) RAW cells were transfected with mouse full-length Ido2 (RAW-Ido2) or empty vector (RAW-MOC). A representative microscopic image of GFP in RAW-MOC or RAW-Ido2 cells is shown. (**b**) The expression of Ido2 mRNA in RAW-MOC or RAW-Ido2 cells was determined by RT-PCR. (**c**) The expression of Ido2 protein in RAW-MOC or RAW-Ido2 cells was determined by western blot. (**d**) Functional Ido2 activity level was determined by measuring the concentrations of Trp and its metabolite, KYN in RAW- Ido2 cells using HPLC (*n* = 4 each). (**e**) The concentration of cytokines was examined in the conditioned medium of RAW-MOC and RAW-Ido2 cells (*n* = 4 each). Data are presented as the means ± SD. *p < 0.05 by one-way ANOVA.

To elucidate the molecular mechanisms controlling Ido2 role in cytokine production, RAW cells were transfected with a GFP-tagged mouse full-length Ido2 (RAW-Ido2) or an empty vector (RAW-MOC) (Fig. 3a). Several RAW-Ido2 clones were established, and Ido2 expression was confirmed by RT-PCR and western blotting (Fig. 3b,c). Increased KYN levels were observed in conditioned medium from RAW-Ido2 cells (Fig. 3d). To confirm the effects of Ido2 on cytokine production, cytokine levels (G-CSF, MCP-1, and MIP-1b) were measured in the conditioned medium of RAW-Ido2 cells. Because RAW cells secreted these cytokines in conditioned medium without stimulus, we examined the levels of G-CSF, MCP-1, and MIP-1b. The production of these cytokines was lower in the conditioned medium of RAW-Ido2 cells than in the conditioned medium of RAW-MOC cells (Fig. 3e).

### Ido2 inhibits cytokine signalling

**Figure 4:**
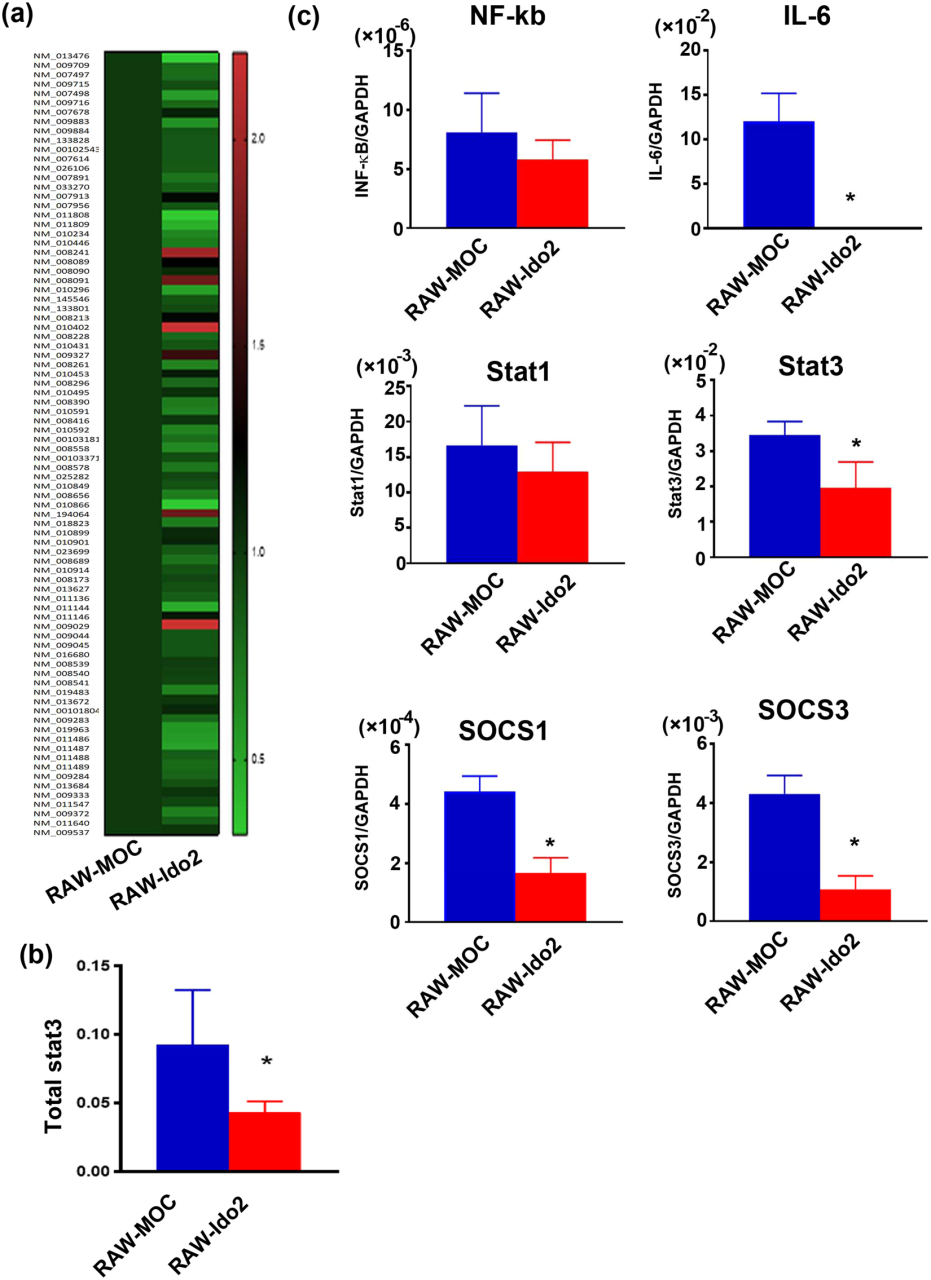
Ido2 inhibits the cytokine signalling. (**a**) Heat maps representing the relative expression of the signalling pathways in RAW-MOC and RAW-Ido2 cells. (**b**) The protein expression of stat3 in RAW-MOC and RAW-Ido2 cells. (**c**) qRT-PCR analysis of LPS induced and stat3-relative gene expression in RAW-MOC and RAW- Ido2 cells. Data are presented as the mean ± SD (*n* = 3 each). *p < 0.05.

**Figure 5:**
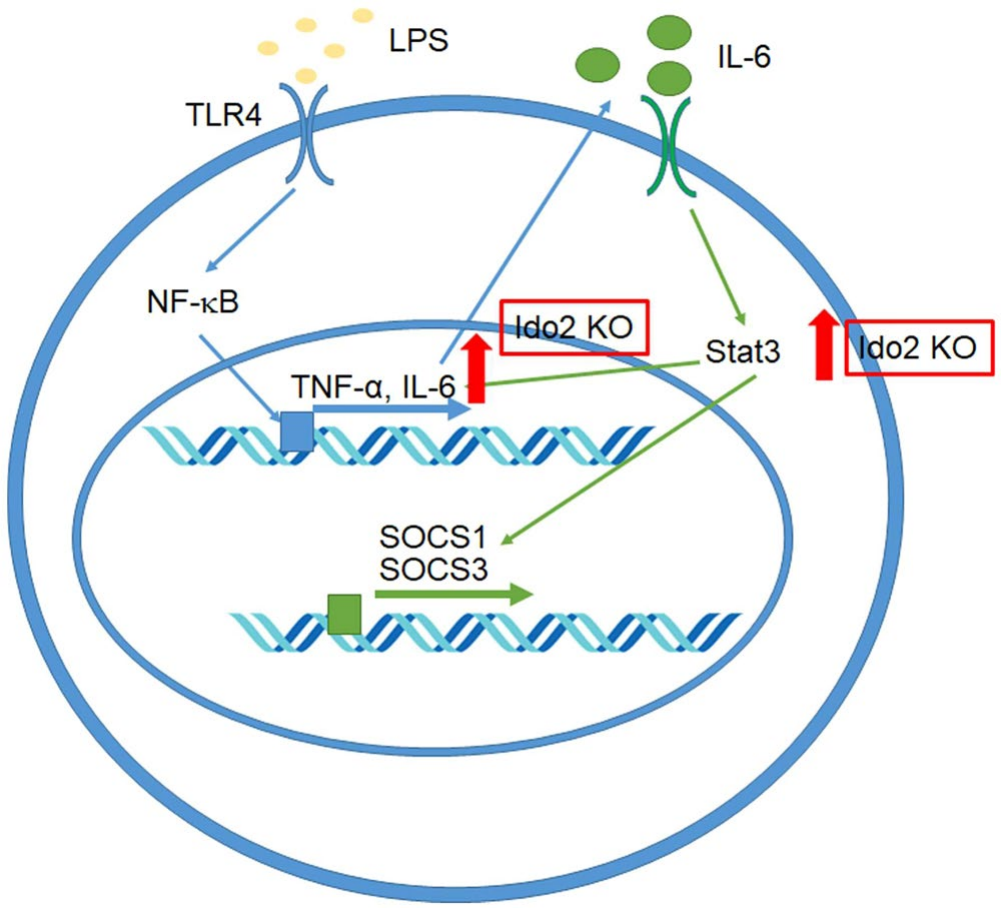
Schematic overview of Ido2 functional role. First, LPS binds to TLR4 receptor and stimulates early cytokine expression via NF-κB. These cytokines, when released, induce stat1 and stat3 activation. Ido2 gene affects IL-6/stat3 signalling after LPS treatments.

We next investigated the roles of Ido2 in cytokine signal transduction in RAW-Ido2 cells. The mouse transcription factors RT² profiler PCR array profiles the expression of 84 genes. The array includes transcription factors that are involved in cytokine, chemokine and growth factor signalling. Array analysis revealed that Ido2 affected stat3 expression (Fig. 4a and Supplemental Table 1). Ido2 overexpressing RAW cells showed decreases in the expression levels of the stat3 protein (Fig. 4b). There was no difference in NF-κB and stat1 expression, but IL-6, SOCS1 and SOCS3 expression decreased in RAW-Ido2 cells compared with RAW-MOC cells (Fig. 4c). These data showed that Ido2 modulates IL-6/stat3-related signal transduction (Fig. 5).

## Discussion

The present study showed that Ido2 is an important molecule in the regulation of cytokine signal transduction pathways, especially IL-6/stat3 signalling. In the LPS-induced endotoxin shock model, Ido2 KO mice were more sensitive to LPS-induced lethality than were the WT mice. LPS-induced cytokine production increased in the macrophages of Ido2 KO mice. LPS is the major outer surface membrane component present in almost all Gram-negative bacteria, and LPS acts as an extremely strong stimulator of innate or adaptive immunity. LPS binds to CD14 and TLR4 and triggers the production of inflammatory cytokines and bioactive molecules that are pivotal in the pathophysiology of sepsis^24^. Sepsis is one major cause of systemic inflammatory response syndrome, which sometimes leads to host death^25^. Among immune cells, macrophages are essential for the initiation of septic disease, as they release various cytokines and chemokines, such as TNFα, IL-6 and MCP-1^26,27^. These cytokines cause multiple organ failures that can result in a high mortality rate. Interestingly, we found a remarkably higher level of IL-6 production in Ido2 KO mice. Among the various cytokines tested, IL-6 is a key mediator of the inflammatory response of sepsis. Pathological overproduction of IL-6 is found in patients with sepsis and correlates with disease mortality^28,29^. The results from the present study confirmed previous reports that IL-6 production was the key cytokine in LPS-induced endotoxin shock model, leading to high mortality after LPS treatment in Ido2 KO mice.

The relationship between the pathophysiology of sepsis and Trp degradation enzymes, such as tryptophan 2,3-dioxygenase 2 (TDO2), Ido1 and Ido2, are reported. Jung *et al*. showed that Ido1 has a protective effect in a sepsis model by controlling the cytokine balance^30^. On the other hand, TDO2 deletion enhances toxicity in sepsis. Bessede *et al*. showed that susceptibility to endotoxaemia was greatly increased in AhR (Aryl hydrocarbon receptor) knockout and TDO2 KO mice compared to WT controls, with limited (Ido1) or no (Ido2) effects of either indoleamine 2,3-dioxygenase deficiency^31^. Our data showed that Ido2 deletion also enhances the toxicity in LPS induced endotoxin models. Further study is needed to clarify the relationship between the pathophysiology of sepsis and Trp degradation enzymes, but our data suggested that Ido2 has an important function in LPS induced endotoxin models.

LPS induces cytokine production through TLR4 and NF-κB signalling activation. Ido2 KO did not affect the NF-κB signal transduction pathway but did influence the stat3 signal transduction pathway. Stat3 is a STAT family member, which is activated in response to IL-6^32^. IL-6 regulates its target genes by binding to its respective receptor complex, thereby activating tyrosine kinases of the Janus family, followed by tyrosine phosphorylation, dimerisation and the nuclear translocation of stat3^33^. These inflammatory cytokines are negatively regulated by SOCS proteins^34^, a family of intracellular proteins that have emerged as key physiological regulators of cytokine responses, including those that regulate the immune system^35^. Interestingly, Ido2 overexpression inhibited SOCS1 and SOCS3 mRNA expression. The expression of SOCS1 and SOCS3 was also regulated by IL-6/stat3 signalling. Our data showed that the Ido2 gene contributes to cytokine production and degradation through IL-6/stat3 signalling.

Under normal conditions, the level of cytokines present is almost the same in WT and Ido2 KO mice. However, once LPS stimulation occurs, cytokine production becomes activated at high levels in Ido2 KO mice. These data suggest that Ido2 is an important regulator of cytokine signalling. Indeed, cytokine production is tightly regulated via a series of beautifully orchestrated pathways. Deregulated cytokine production is implicated in several disease states, ranging from chronic inflammation to allergies^36^. Our data indicate that the deletion of Ido2 causes the degradation of regulated cytokine production pathways. Recently, a proinflammatory role for Ido2 has been reported, showing that Ido2 KO mice show a delayed onset or reduced severity of arthritis^21^. Furthermore, Ido2-deficient B cells lack the ability to upregulate the co-stimulatory marker CD40, suggesting that Ido2 acts at the interface between T cells and B cells to modulate T cell potency to promote autoantibody production^23^. Although the reason for the discrepancy between our results and previous studies is not yet understood, Ido2 plays an important role in cytokine production in macrophages and in antibody production in B cells.

Ido1 is induced by proinflammatory cytokines, such as IFN-γ, and catalyses the degradation of Trp to KYN. Although the mechanism of Ido2 induction remains unknown, some reports have shown that Ido2 mRNA expression is upregulated in response to IFN-γ in some cell lines^37,38^, DCs and mesenchymal stem cells^39^. On the other hand, in an experimental cerebral malaria model that has high levels of circulating IFN-γ, Ido2 mRNA is not upregulated^18^. In the present study, macrophages were shown to induce Ido2 expression after LPS treatment, but further study is needed to elucidate the mechanism of Ido2 induction in various conditions.

Macrophages of Ido2 KO mice enhanced cytokine production induced by LPS. However, the macrophages of Ido1 KO mice treated with LPS failed to change their cytokine production. Previous reports showed that Ido2 has a low affinity for Trp in a cell-free assay, resulting in low catalytic efficiency compared with Ido1^40–42^. For this reason, Ido1 KO may increase KYN concentration to a greater extent compared with Ido2 KO in mice. If the KYN concentration is the key factor governing cytokine production, Ido1 KO mice may induce greater cytokine production compared with Ido2 KO mice. The present data suggest that the mechanisms controlling immune modulation are different for Ido1 and Ido2. Although Ido1 plays an important role in the production of metabolites in the KYN pathway, Ido2 affects cytokine signal transduction. Even though further study will be needed to elucidate the precise mechanism, our data suggest that Ido2 controls the cytokine signal transduction pathway without changes to the KYN concentration or homeostatic KYN levels. Therefore, Ido2 is a negative regulator of cytokine signalling in the immune system and is important for maintaining cytokine homeostasis.

## Methods

### Animals

All mice were mature males, 6–8 weeks old. Ido2 KO mice on a C57BL/6 background were obtained from the Knockout Mouse Project (KOMP, CA, USA). Mice that were homozygous null (Ido2 KO) by targeted disruption of the Ido2 gene were selected from the offspring of heterozygous-homozygous matings by genotyping with tail DNA PCR. Ido1 KO mice on a C57BL/6 background were obtained from Jackson Laboratory (Bar Harbor, ME, USA). WT C57BL/6 N mice were purchased from Charles River Laboratories (Yokohama, Japan). Animals were housed in the animal facilities of Fujita Health University Graduate School of Medicine under specific pathogen-free conditions, maintained at 25 °C on a 12 h light/dark cycle (lights on at 08:00), and had free access to food and water. The protocol for all animal experiments was approved by the Animal Experimentation Committee of Fujita Health University Graduate School of Medicine. Procedures involving mice and their care conformed to international guidelines, as described in *Principles of Laboratory Animal Care* (National Institutes of Health publication 85-23, revised 1985).

### Induction of LPS-induced Endotoxin Shock

WT and Ido2 KO mice (body weight 20–23 g) were intraperitoneally injected with 15 mg/kg of LPS from *Salmonella enterica* (Sigma-Aldrich, MO, USA), and their survival was monitored daily for 7 days. To obtain samples, the animals were anaesthetized and humanely sacrificed at the indicated times.

### Separation of Peritoneal Macrophages and T Cell

Two and a half millilitres of 3% thioglycollate medium was intraperitoneally injected into Ido2 KO mice or WT mice. Four days after injection, cells were collected from the peritoneal cavity. Peritoneal macrophages were enriched using the EasySep Negative Mouse Monocyte Kit (Stemcell Technologies Inc., MA, USA). T-cells were isolated from spleen in Ido2 KO mice or WT mice using the EasySep Negative Mouse T Cell Kit (Stemcell Technologies Inc.) according to the manufacturer’s instructions.

Peritoneal macrophages and T-cells were incubated for 24 h in 12-well, flat-bottom plates in RPMI-1640 with or without 100 ng/ml LPS (Sigma-Aldrich, MO, USA).

### Measurement of Kynurenine Pathway Metabolites

We used high-performance liquid chromatography (HPLC) to measure the Trp and kynurenine pathway metabolite, KYN, in conditioned medium and mouse serum. Specifically, we used a TSKgel ODS-100 V 3 µm 4.6 mm (ID) × 15 cm (L) column with a spectrophotometric detector (Shimazu, Tokyo, Japan), as described in our previous reports^43,44^.

### Cytokine Assays

To measure the cytokines in mouse serum or conditioned medium, we used a Bio-Plex multiplex assay system (Bio-Rad Technologies, CA, USA). The specific cytokines identified with this approach were IL-1α, IL-2, IL-3, IL-6, IL-10, MCP-1, MIP-1α, MIP-1β, RANTES and TNF-α.

### Stat3-relative protein expression and phosphorylation levels of stat3

The phosphorylated stat3 levels and stat3 protein levels were examined using a stat3 (pY705) and total stat3 ELISA Kit (ab126459) (Abcam, Cambridge, UK). A sandwich ELISA system is an *in vitro* enzyme-linked immunosorbent assay for the measurement of phospho-stat3 (Tyr705) and total stat3. Each experiment was performed according to the manufacturer’s protocols.

### Histopathology

Tissues were fixed in 10% formalin in phosphate-buffered saline overnight and then embedded in paraffin. Sections (thickness, 4 μm) were used for haematoxylin and eosin (H&E) and Ido2 immunohistochemical staining. Anti-Ido2 antibody (ab214214) (Abcam, Cambridge, UK) and simple histofine (Nichirei Corporation, Tokyo, Japan) were used for Ido2 staining.

### Cell Culture

A murine macrophage cell line (RAW) consisting of 264.7 cells that originated from the American Type Culture Collection was maintained in RPMI-1640 medium (Nissui Co., Ltd., Tokyo, Japan) supplemented with 10% FCS, at 37 °C in a humid atmosphere with 5% CO_2_. Full-length mouse Ido2 cDNA was subcloned into an N-terminal Venus-tagged CAGGS expression vector. Mouse Ido2 cDNA was transfected into RAW cells (RAW- Ido2) with lipofectamine (Life Technologies, CA, USA), and Ido2 expression was confirmed by mRNA and protein levels.

### RNA Extraction and Quantitative Real Time PCR

Total RNA was extracted from cell lines with ISOGEN (Nippongene, Tokyo, Japan) and reverse transcription-PCR was performed using Revetra Ace Kits (Toyobo, Tokyo, Japan). Real time PCR was performed using the EvaGreen PCR Master Mix (Bio-Rad Technologies, CA, USA). The mRNA expression of target genes was normalised to GAPDH. The primer sequences were as follows: GAPDH, sense 5ʹ-TGCACCACCAACTGCT-3ʹ, antisense 5ʹ-GGCATGGACTGTGGTC-3ʹ; Ido1, sense 5ʹ-CCCACACTGAGCACGG-3ʹ, antisense 5ʹ-TTGCGGGGCAGCACCT-3ʹ; Ido2, sense 5ʹ-GCCCAGAGCTCCGTGC-3ʹ, antisense 5ʹ-TGGGAAGGCGGCATGT-3ʹ.

### Western blot

Cell extracts were prepared in lysis buffer [100 mM Tris-HCL (pH 8.0), 100 mM NaCl, 0.5% NP40, and 0.2 mM phenylmethane sulfonylfluoride] and centrifuged for 5 min at 1800 g at 4 °C. The supernatants collected for protein analysis were lysed with SDS-PAGE sample buffer, sonicated and boiled. Protein samples were separated by one-dimensional SDS-PAGE, transferred onto a PVDF membrane and blotted with Ido2 (ab214214) from Abcam (Cambridge, UK). The immunoblot signals were normalized to β-actin (PM053) from MBL (Tokyo, Japan).

### Gene Array Analysis

Total RNA was isolated and reverse transcribed. qPCR was performed using Mouse Transcription Factors RT² Profiler PCR Array profile (QIAGEN, Hilden, Germany) (QIAGEN) containing primers for 84 genes. We assayed the gene expressions of the 84 key mouse signal transduction related genes.

### Statistical Analysis

GraphPad Prism v.7 software was used for statistical analyses. The survival rate of the mice was analysed using the Kaplan–Meier method. Statistically significant differences between groups were determined using Student’s t-test or one-way analysis of variance. A *p* value < 0.05 was considered statistically significant, and 95% confidence intervals are indicated within figures.

## Electronic supplementary material

Dataset 1
