# Supplementary material for: Lipopolysaccharide shock reveals the immune function of indoleamine 2,3-dioxygenase 2 through the regulation of IL-6/stat3 signalling
Source: Sci Rep. 2018 Oct 29;8:15917. doi: 10.1038/s41598-018-34166-4 (PMC6206095; doi:10.1038/s41598-018-34166-4)
Supplement: Supplementary file 1 — Dataset 1 [file 41598_2018_34166_MOESM1_ESM.docx]

**Lipopolysaccharide shock reveals the immune function of indoleamine 2,3-dioxygenase 2 through the regulation of IL-6/stat3 signalling**

Yasuko Yamamoto, Wakana Yamasuge, Shinjiro Imai, Kazuo Kunisawa, Masato Hoshi, Hidetsugu Fujigaki, Akihiro Mouri, Toshitaka Nabeshima and Kuniaki Saito.

**Inventory of Supplemental Information**

**Supplemental Figures (Figure S1-Figure S4)**

**Legends to Supplemental Figures**

**Supplemental Table 1**

Supplemental Figure 1

Immunohistochemical image of Ido2 in WT mice treated with LPS.

Ido2 protein expression in WT mice 24 h after LPS administration was assessed by immunohistochemistry. Illustrative images from formalin-fixed paraffin-embedded tissue from WT control and LPS-treated mice. A positive signal for Ido2 was detected in the immune cells. Left panel, low-power field. Right panel, high-power field. Scale bar, 200 μm.

Supplemental Figure 2

Ido1 and Ido2 expression in WT macrophages treated with LPS.

The mRNA expression of Ido1 (a) and Ido2 (b) in the peritoneal macrophages of WT mice treated with LPS for 24 h (*n* = 3 each). Data are normalised to GAPDH expression. Data are presented as the means ± SD. **p* < 0.05 by Student's *t* test.

Supplemental Figure 3

Cytokine production by T cells in WT and Ido2 KO mice.

The cytokine level in conditioned medium was detected in T cells of WT and Ido2 KO mice treated with LPS (*n* = 4 each). Data are presented as the means ± SD.

Supplemental Figure 4

Cytokine production in macrophages in WT and Ido1 KO mice.

The cytokine level in conditioned medium was measured in macrophages of WT and Ido1 KO mice treated with LPS (*n* = 4 each). Data are presented as the means ± SD.
